# Evaluation of an alternative heterotopic transplantation model for ovarian tissue to test pharmaceuticals improvements for fertility restoration

**DOI:** 10.1186/s12958-022-00910-9

**Published:** 2022-02-19

**Authors:** Carmen Terren, Jules Bindels, Michelle Nisolle, Agnès Noël, Carine Munaut

**Affiliations:** 1 Laboratory of Tumor and Development Biology, GIGA-Cancer, University of Liège, Tour de Pathologie , Site Sart-Tilman, Building 23/4, Avenue Hippocrate, 13, 4000 Liege, Belgium; 2 Department of Obstetrics and Gynecology, Hôpital de La Citadelle, University of Liège, B-4000 Liège, Belgium

**Keywords:** Ovarian tissue transplantation, Heterotopic transplantation, Animal model, Fertility restoration

## Abstract

**Background:**

Ovarian tissue cryopreservation and transplantation (OTCTP) is currently the main option available to preserve fertility in prepubertal patients undergoing aggressive cancer therapy treatments. However, a major limitation of OTCTP is follicle loss after transplantation. The mouse is a model of choice for studying ovarian function and follicle development after ovarian tissue grafting in vivo. In these mouse models, ovarian tissue or ovaries can be transplanted to different sites. Our aim was to evaluate a new alternative to heterotopic transplantation models that could be useful to test pharmaceutical improvement for ovarian grafts after OTCTP.

**Methods:**

Slow frozen murine whole ovaries were transplanted into the mouse ears (between the external ear skin layer and the cartilage). Ovarian transplants were recovered after 3, 14 or 21 days. Grafts were analyzed by immunohistochemistry and follicle density analyses were performed.

**Results:**

An increase of ovarian vascularization (CD31 and Dextran-FITC positive staining), as well as cellular proliferation (Ki67 staining) were observed 3 weeks after transplantation in comparison to 3 days. Fibrosis density, evaluated after Van Gieson staining, decreased 3 weeks after transplantation. Furthermore, transplantation of cryopreserved ovaries into ovariectomized mice favored follicle activation compared to transplantation into non-ovariectomized mice.

**Conclusion:**

The present study indicates that surgical tissue insertion in the highly vascularized murine ear is an effective model for ovarian grafting. This model could be helpful in research to test pharmaceutical strategies to improve the function and survival of cryopreserved and transplanted ovarian tissue.

## Background

Cryopreservation of ovarian tissue followed by its auto-transplantation is currently the main option to preserve the fertility of prepubertal patients or when oncological care is urgent [1, 2]. However, this technique has certain limitations, including follicular loss immediately after grafting, possibly due to slow neovascularization, apoptosis [3, 4] and/or massive follicular recruitment, which is also known as follicular burnout [5]. Primordial follicle overactivation leads to a depletion of the ovarian reserve and thus to a reduced lifetime of the transplant [5]. Experimental *in vivo* models are of high value in this research field in order to test pharmacological strategies to limit transplantation-induced follicle loss and therefore to increase graft lifetime as well as the chances of pregnancies. In the literature, a number of transplantation sites have been investigated, including grafting intraperitoneally [6, 7], into the ovarian bursa [8, 9], under the kidney capsule [10, 11], intramuscularly [12] or subcutaneously [13, 14]. A good transplantation site promotes fast revascularization of ovarian tissue, limiting the duration of ischemia and hence ovarian damage. The ovarian bursa is an interesting transplantation site as it is highly vascularized and natural pregnancies after murine ovarian transplantation are possible [8, 14, 15]. However, grafting beneath the murine ovarian bursa has several disadvantages compared to other sites. Indeed, only small tissue pieces can be inserted into the bursa via a small slit under microscopic control. In a recent study, the ovarian bursa site showed however the best ovarian tissue quality compared to subcutaneous graft sites [14]. This could be explained by the fact that in the subcutaneous site, the environment, pressure changes and temperature could influence ovarian quality. However, the subcutaneous graft site involves a simple surgical procedure and allows for external follow-up of follicular growth [16].

The intraperitoneal site may be an equivalent to the orthotopic graft site used in clinic [17]. Indeed, it provides a favorable environment for follicular development [16]. However, invasive surgery is necessary.

Our aim was to evaluate an alternative to heterotopic and/or other classical transplantation models for ovarian grafts through an adaptation of the “ear sponge assay” which was previously set up to study angiogenesis and lymphangiogenesis [20, 21]. This model involves ovarian tissue transplantation into the mice ear (between the skin layer and the cartilage). The first important advantage is to be less invasive than conventional models. Secondly, the transplantation site is highly vascularized. This feature is of high value in the case of ovarian grafts since a major follicle loss has been documented during the avascular ovarian transplantation [8, 18, 19, 22–24]. Finally, transplanted tissues remain easily accessible for subsequent therapeutic local treatments to limit follicle loss observed after transplantation of cryostored ovarian tissue.

We therefore evaluated this model by studying graft revascularization and survival after cryopreservation and transplantation.

## Materials and methods

### Experimental design

Mice were bred and maintained within the accredited Mouse Facility and Transgenics GIGA platform of the University of Liège (Belgium). The first experiment consists of the model validation. Therefore, slow frozen (SF) ovaries from SCID mice (6–10 weeks old) were transplanted ectopically into the ear of SCID mice (12–14 weeks old, not ovariectomized) for either 3 days or 3 weeks (*n* = 4–5 ovaries per group with one graft per mice). We also performed a comparison of follicle development of SF ovaries from BALB/c mice (7 weeks old) transplanted into ovariectomized or non-ovariectomized SCID mice (7-11 weeks old) with transplants recovery after 2 weeks (*n* = 11–12 ovaries per group with one graft per mice). Transplanted ovaries from all experiments were fixed in 4% formaldehyde for histological assessment. The Animal Ethics Committee of the University of Liège approved this study (#1934) and all experiments were performed in accordance with relevant guidelines and regulations.

### Ovariectomy, slow freezing (SF) and thawing procedure

This procedure was previously described [25].

### Transplantation procedure

**Fig. 1:**
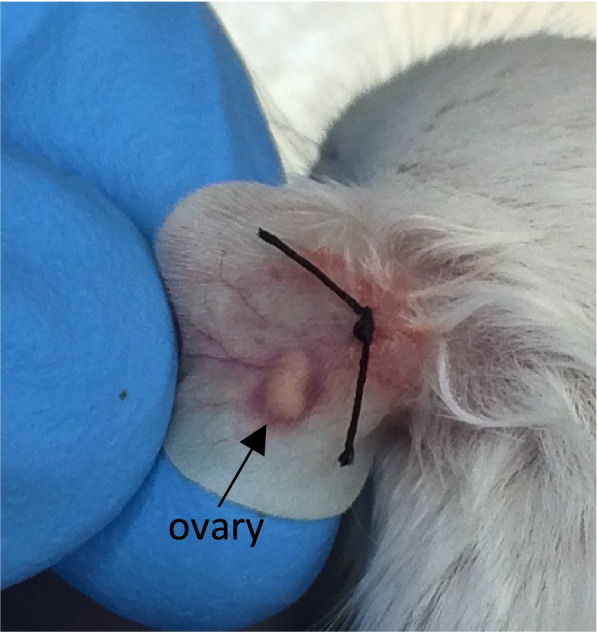
Photograph of the place where ovaries were grafted. A horizontal incision was performed in the basal, external and central part of the mouse ear and the external mouse ear skin layer was smoothly detached from the cartilage with thin forceps. The ovary was introduced inside the hole, between the external mouse skin layer and the cartilage. A suture point was made to close the skin incision

Mice were anesthetized with ketamine hydrochloride (100 mg/kg body weight) and xylazine (10 mg/kg body weight) by intra-peritoneal injection and a small horizontal incision was performed in the basal, external, and central parts of the ear and the external mouse ear skin layer was smoothly detached from the cartilage with thin forceps. The cryopreserved ovary (whole ovary) was introduced inside the hole, between the external mouse skin layer and the cartilage (Fig. 1). A suture point was made to close the skin incision.

### Histological assessment

Intravenous injection of 200 μl dextran-fluorescein isothio-cyanate (FITC, 2.5 mg/ml in PBS) was given to mice before sacrifice and then the skin of the ear was cut around the transplanted ovary. Ovaries fixed in 4% formaldehyde were paraffin-embedded and serially sectioned (5 μm sections). The immunohistochemical detection of vascular endothelial cells (CD31) and functional blood vessels (dextran-FITC) was performed using specific primary antibodies (rabbit anti CD31 1/200 during 1 h (Abcam, Cambridge, UK) and anti-fluorescein-POD/HRP during 30 min (Roche, Basel, Switzerland). Fibrosis and nucleus density were analyzed thanks to Van Gieson staining. Apoptosis and cell proliferation in the ovaries were evidenced by immunostaining of caspase-3 (1/300 overnight at 4 °C, Cell Signaling, Danvers, USA) and Ki67 (1/100 during 1 h, Abcam, Cambridge, UK), respectively.

Density of immunostaining was determined by computer-assisted image analysis as previously described [25]. For follicle quantification, hematoxylin and eosin sections were analyzed by light microscopy for the presence of primordial, primary and secondary or more mature follicles based on morphological classification of mouse follicles [26]. The follicular densities (number/mm^2^) were calculated after manually outlining the ovarian surface (NDP view software-NDP.view2 Viewing software U12388-01, Hamamatsu Photonics K.K., Japan).

### Statistics

GraphPad Prism (GraphPad, San Diego, CA, USA) was used for statistical analyses. All data are presented as means ± SEM. The Mann Whitney test was applied and *p*-value < 0.05 was considered statistically significant.

## Results

### Grafts are revascularized 3 weeks after transplantation with an increased cell proliferation

**Fig. 2:**
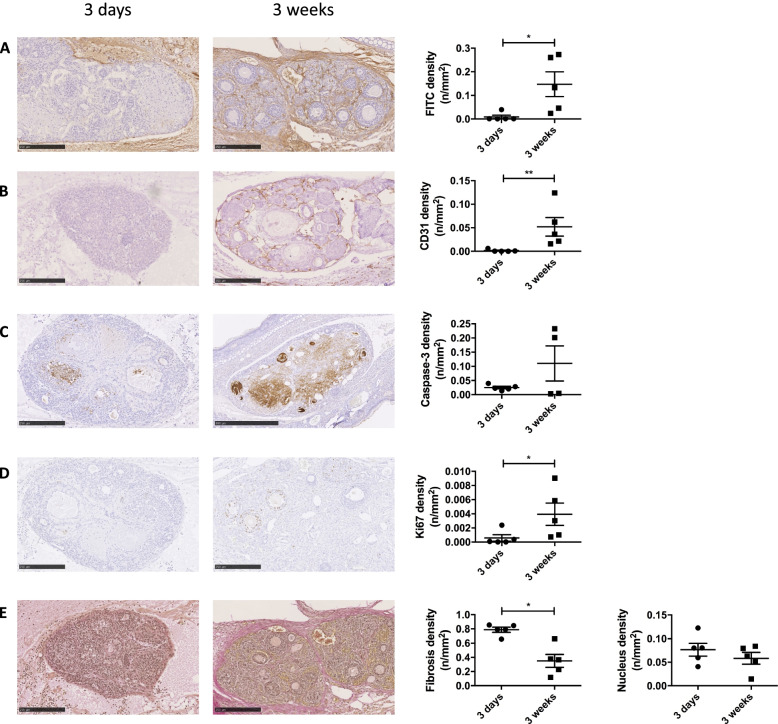
Vascularization and proliferation assessment. Slow frozen (SF) ovaries from SCID mice (6–10 weeks old) were transplanted to SCID mice (12–14 weeks old, not ovariectomized) for either 3 days or 3 weeks. Representative images and computer-assisted quantification (mean ± SEM) of (**A**) FITC staining, (**B**) CD31 staining, (**C**) caspase-3 staining, (**D**) Ki67 staining and (**E**) Van Gieson staining with fibrosis and nucleus staining density. *n* = 4–5 ovaries per group. * *p* ≤ 0.05; **p < 0.01

A significant increase of vascular endothelial cells (CD31 staining) and functional blood vessels (FITC staining) was observed after 3 weeks of transplantation compared to 3 days (Fig. 2A-B). No modulation in apoptosis (Fig. 2C) was observed. Cell proliferation, as detected by the Ki67 staining quantification was increased after 3 weeks (Fig. 2D). Furthermore, a decrease in fibrosis density was observed after 3 weeks of transplantation compared to 3 days (Fig. 2E) but no modulation in nuclear density was observed (Fig. 2E).

### Follicle activation is more pronounced after ovarian transplantation into ovariectomized compared to non-ovariectomized mice

**Fig. 3:**
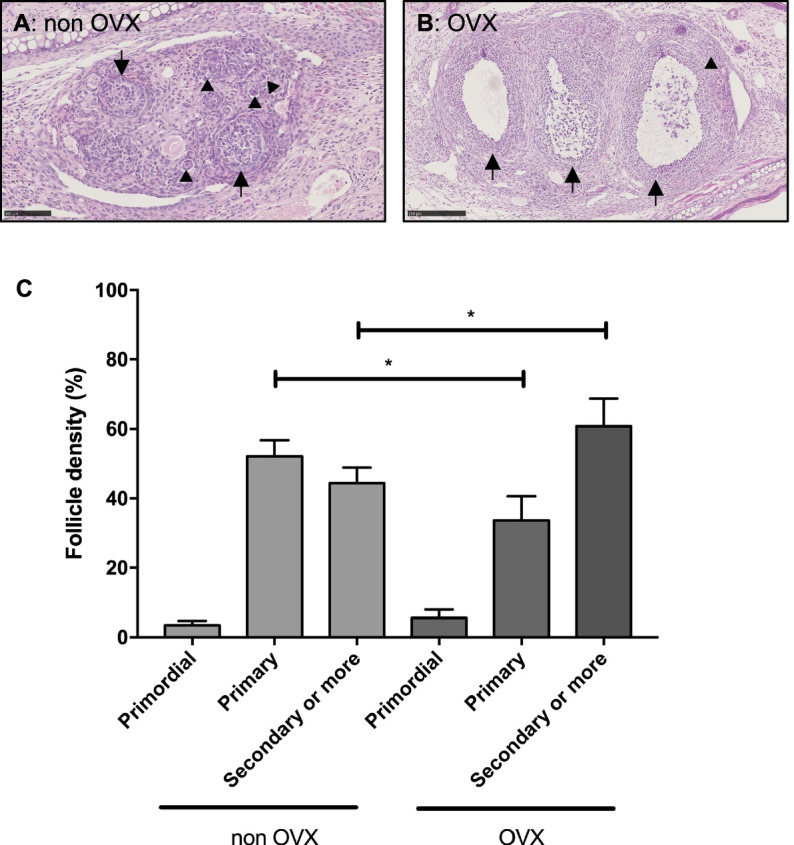
Follicle development after transplantation into ovariectomized or non-ovariectomized mice. Transplantation of slow frozen (SF) ovaries from BALB/c mice (7 weeks old) into SCID mice (7-11 weeks old) for 2 weeks. Representative images of hematoxylin & eosin staining of ovaries transplanted into a non-ovariectomized (**A**) and an ovariectomized (**B**) mice. Arrowheads show primary follicles and arrows show secondary or more mature follicles. (**C**) Follicle density of primordial, primary or secondary or more mature follicles (means ± SEM). *n* = 11–12 ovaries per group. * *p* ≤ 0.05. OVX = ovariectomy

A significant increase of secondary or more mature follicles was observed in ovaries transplanted into ovariectomized mice accompanied with a decrease in primary follicle density compared to non-ovariectomized mice. However, primordial follicle density was similar between the two experimental groups (Fig. 3 A-C).

## Discussion

A number of different ovarian transplantation models exists (e.g., intraperitoneal, under the kidney capsule, into the ovarian bursa, subcutaneous, …). Their main inconvenient is that they are all invasive, with the exception of subcutaneous transplantation sites [8, 9, 11, 16, 18, 19]. Our aim was therefore to develop a simpler and less invasive model, which is in addition easily accessible for local injections of different agents to improve graft health.

First, SF ovaries were transplanted into mice ears for 3 days or 3 weeks. An increase of ovarian vascularization was observed 3 weeks after transplantation compared to 3 days (CD31 and FITC staining). Indeed, hypoxic period was identified during the first 5 days after transplantation followed by gradual oxygenation of the ovarian transplant over the next 5 days [6]. Additionally, nuclear density and apoptosis remained stable through the transplantation time, indicating that cellular death is not increased 3 weeks after transplantation compared to 3 days. A decrease in fibrosis density was observed 3 weeks after transplantation compared to 3 days. However, an increase in fibrosis density was observed when marmoset, bovine or human ovarian tissue was transplanted subcutaneously into immunodeficient mice for 7 days compared to 3 days [19]. This observation determines an advantage of the ear transplantation site compared to the subcutaneous transplantation site.

On the other hand, Ki67 staining increased after 3 weeks of transplantation compared to 3 days. This could indicate follicle activation linked to transplantation. Altogether, these results indicate that this model could be usable as an alternative to invasive models.

In order to determine whether the mice own ovaries have an influence on the transplanted ovaries, a comparison of follicle development of ovaries transplanted into ovariectomized or non-ovariectomized mice has been performed. A significant increase of secondary or more mature follicles was observed in ovaries transplanted into ovariectomized mice accompanied with a decrease in primary follicle density compared to ovaries transplanted into non-ovariectomized mice. One of the likely explanations for the transplantation-induced activation of primordial follicles in ovariectomized mice lies in the absence of the mice own ovaries. In normal physiology, growing follicles maintain the quiescence of primordial follicles via the production of inhibitory factors, such as antiMullerian hormone (AMH) [20]. Furthermore, in a cryopreserved ovary, most of the mature follicles do not resist the cryopreservation process while primordial follicles, which have a low metabolic activity, are more tolerant to cryopreservation [21]. Therefore, in an ovariectomized mice, there is no AMH secretion (neither from the transplanted ovary nor from the removed mice own ovaries). An accelerate follicle activation was therefore observed. Indeed, the absence of growing follicles disrupts the balance between stimulatory and inhibitory factors in the graft, thereby leading to follicle activation [5, 19, 27]. In contrast, when the recipient mice are non-ovariectomized, the mice own ovaries are secreting AMH, acting on the transplanted ovary to limit primordial follicle activation.

## Conclusion

In conclusion, the ear transplant model could be suitable for ovarian tissue transplantation due to the high revascularization rate. Furthermore, transplantation of cryopreserved ovaries into ovariectomized mice favors follicle activation as compared to transplantation into a non-ovariectomized mice. This new model is of particular interest for the testing of various pharmaceuticals strategies to limit follicle loss associated to the avascular ovarian auto-transplantation.

## Data Availability

The data underlying this article will be shared on reasonable request to the corresponding author.

## Abbreviations

OTCTP: Ovarian tissue cryopreservation and transplantation
FITC: Fluorescein isothio-cyanate
AMH: AntiMullerian hormone
SF: Slow freezing
OVX: Ovariectomy
